# Non-monotonic dose-response relationships and endocrine disruptors: a qualitative method of assessment

**DOI:** 10.1186/1476-069X-14-13

**Published:** 2015-02-11

**Authors:** Fabien Lagarde, Claire Beausoleil, Scott M Belcher, Luc P Belzunces, Claude Emond, Michel Guerbet, Christophe Rousselle

**Affiliations:** Risk Assessment Department, French Agency for Food, Environmental and Occupational Health & Safety (ANSES), 14 rue Pierre et Marie Curie, 94701 Maisons-Alfort Cedex, France; Department of Pharmacology and Cell Biophysics, University of Cincinnati, College of Medicine, Cincinnati, OH USA; INRA, Laboratoire de Toxicologie Environnementale, UR 406 A&E, CS 40509, 84914 Avignon Cedex 9, France; BioSimulation Consulting Inc., Newark, DE USA; Université de Rouen, UFR Médecine Pharmacie, Laboratoire de Toxicologie, UR 4651 ABTE, 76183 Rouen Cedex 1, France

**Keywords:** Endocrine disruptors, NMDR, Non-monotonic, Dose-response, Risk assessment, Bisphenol A

## Abstract

Experimental studies investigating the effects of endocrine disruptors frequently identify potential unconventional dose-response relationships called non-monotonic dose-response (NMDR) relationships. Standardized approaches for investigating NMDR relationships in a risk assessment context are missing. The aim of this work was to develop criteria for assessing the strength of NMDR relationships. A literature search was conducted to identify published studies that report NMDR relationships with endocrine disruptors. Fifty-one experimental studies that investigated various effects associated with endocrine disruption elicited by many substances were selected. Scoring criteria were applied by adaptation of an approach previously used for identification of hormesis-type dose-response relationships. Out of the 148 NMDR relationships analyzed, 82 were categorized with this method as having a “moderate” to “high” level of plausibility for various effects. Numerous modes of action described in the literature can explain such phenomena. NMDR can arise from numerous molecular mechanisms such as opposing effects induced by multiple receptors differing by their affinity, receptor desensitization, negative feedback with increasing dose, or dose-dependent metabolism modulation. A stepwise decision tree was developed as a tool to standardize the analysis of NMDR relationships observed in the literature with the final aim to use these results in a Risk Assessment purpose. This decision tree was finally applied to studies focused on the effects of bisphenol A.

## Background

Non-monotonic dose-response (NMDR) relationships are more frequently reported today in experimental studies than they were 10 years ago [1–3]. The endocrine disrupting chemicals (EDCs) are regularly associated with NMDR relationships. Until recently, NMDR relationships were not considered plausible, and thus they were not published, reported, or interpreted as relevant biological phenomena. An increasing number of scientists think that NMDR relationships represent a toxicological reality, but supplementary effort is required to revisit the Paracelsus principle of “*the dose makes the poison*”. Consequently, it is important to avoid rejecting studies merely because they do not match the classic toxicological concept of monotonicity. Before affirming that such phenomenon exists in a study, a screening analysis of individual NMDR profile is required to reveal better understanding of the underlying biological mechanism (s) involved and support plausibility of the observed NMDR.

The term “NMDR” describes a dose-response relationship characterized by a curve whose slope changes direction within the range of tested doses. Non-monotonicity represents a challenge to fundamental concepts in toxicology and risk assessment. Indeed, environmental risk assessment approaches used by regulatory agencies around the world were developed on the basis of a methodology published by the National Academy of Sciences [4]. For the hazard characterization step, it is generally accepted that once detectable, a response of an organism to a toxicant increases proportionally with the level of exposure until reaching an upper-limit or maximal-effect level (E_max_) beyond which increasing toxicant dose will not increase the response (known as a monotonic dose-response). To assess the dose-response relationship of a chemical, several doses are typically tested to define the no observed adverse effect level (NOAEL) and/or the lowest observed adverse effect level (LOAEL). The NOAEL is considered a conservative default threshold below which a chemical is not expected to induce adverse effects, irrespective of the dose [5].

In contrast to this well-accepted hyperbolic or curvilinear dose-response relationship, which when plotted as toxicant effect (ordinate) and the logarithm (log10) of dose (abscissa) results in a sigmoidal shaped curve, a NMDR relationship may present as a bell-shaped profile. This profile, also called inverted-U shape, is characterized by responses at intermediate dose (s) and a decreased response or no response observed at low- and high-exposure levels. Also observed in the literature are U-shaped profiles with the highest responses at low- and high-exposure levels. In such cases, the standard notion of threshold, as defined by the NOAEL concept, is not inclusive of all potentially harmful effects. As clearly stated by Vandenberg et al. [3], “if a non-monotonic relationship occurs between the doses tested in traditional toxicology studies (i.e. the NOAEL or NOEL) and the calculated “safe” or reference dose, this would still have serious implications for risk assessment”. Thus, the standard approaches used for setting safe human and environmental exposure levels by extrapolation from high dose testing might not be applicable in case of NMDR profiles. There is a class of toxicants (i.e., EDCs) for which NMDR relationships, have been experimentally described with relative high frequency compared to other chemicals [6–11]. The EDCs act through several modes of action to cause effects on sensitive tissues. As a result, EDCs can commonly act on many physiological systems in addition to endocrine tissues. Effects observed in sensitive target tissues can also be caused by exposure-related changes in mechanisms governing normal negative feedback regulation of endocrine tissues and hormone secretion; such effects may also influence the dose-response relationship depending on exposure duration. With increasing awareness, more studies are being specifically designed to address whether the dose-response relationships for EDCs and other chemicals are described with an NMDR curve. As a result, NMDR relationships are being reported with an increasing frequency, which may have consequences for risk assessment. As reported by Vandenberg et al. [3], regardless of mechanistic underpinnings of each NMDR, their existence alone challenged traditional means of risk assessment.

The work described in this manuscript aimed to (1) perform a focused literature review, (2) extract a representative set of observed NMDR relationships for some EDCs and (3) use that information to develop a methodology to assess whether such a reported dose-response relationship is sufficiently reliable in order to be used in risk assessments. The screening analysis method evaluating the likelihood and the plausibility of the biological mechanisms involved was applied for each identified NMDR. Then, a stepwise decision tree was developed as a tool to standardize analysis for data reliability of observed *in vivo* NMDR relationships for risk assessment.

## Methods

### Screening analysis of the literature

A literature review was conducted by selecting putative EDCs for which one or more NMDR profiles were observed. Many keywords related to EDCs (i.e., hormones or exogenous substances for which a general consensus exists for their endocrine properties) and NMDR relationships were used to search the PubMed database, with a data-lock point of January 2012. The keywords used to screen EDCs were bisphenol* OR phthalate* OR paraben* OR phenol* OR PCB (polychlorinated biphenyls)* OR diethylstilbestrol OR estrogen* OR estradiol*. The keywords used to screen NMDR relationships were hormesis OR hormetic OR non-monotonic OR inverted-U OR U-shape* OR J-shape* OR bell-shape* OR biphasic. All published articles outlining an NMDR relationship with a tested compound were selected.

To determine whether the existence of an NMDR relationship was supported by the available data, a qualitative methodology was applied by considering the statistical strength and the biological plausibility of each reported NMDR profile. To assess the statistical strength of NMDR profile, an appropriate statistical analysis based on individual results demonstrating the non-monotonic nature of each dose-response relationship is essential. There were cases in which a proper statistical analysis was not performed by the authors and individual data were poorly reported. Therefore, in these cases, it was difficult to confirm *a posteriori* that the observed relationships were statistically significant for a non-monotonic behavior. For those cases, specific scoring criteria were applied to assess the strength of the NMDR relationships. This scoring criteria was derived from the identification criteria proposed by Calabrese and Baldwin [12] for hormesis (a specific type of NMDR profile).

The biological plausibility of NMDR relationships reported as significant, or scored to have sufficient strength, were evaluated by considering whether mechanistic explanations were proposed or demonstrated for the observed dose-response relationship. If the proposed hypotheses were supported by experimental evidence, then the biological plausibility was considered as reinforced.

### Stepwise decision tree

**Figure 1:**
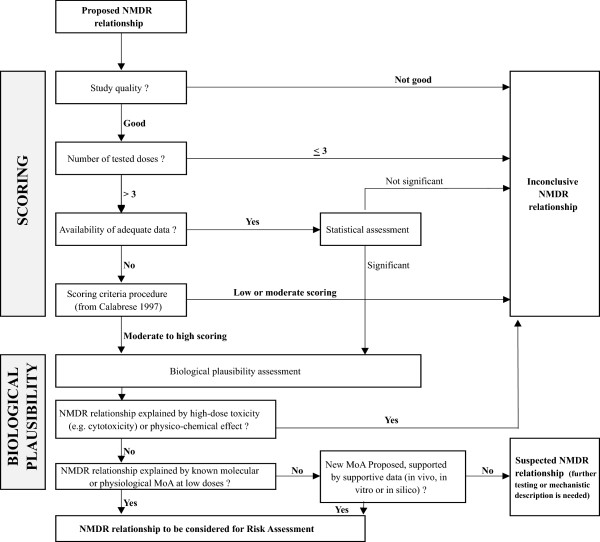
**Decision tree describing the methodology for evaluating the plausibility of an NMDR relationship.**

A stepwise decision tree was developed (Figure 1) to assess whether observed NMDR profiles for EDCs could be used in the context of risk assessment. Using this approach, data is first qualified using the Klimisch score as an ancillary approach to assess quality of the study and the experimental data [13]. If the quality is identified as Category 3 or 4, then the study will be characterized as an inconclusive NMDR relationship.

If the quality of the study is Category 1 or 2, then we will proceed to the second step and consider the number of tested doses analyzed in the study. If less than or equal to three (inclusive of the negative control), the number of doses will be judged to be insufficient to establish a dose-response relationship and the study is characterized as an inconclusive NMDR relationship.

For studies with more than three doses, the third step depends on the availability (or not) of adequate experimental data to continue the plausibility assessment of a NMDR. To assess the strength of NMDR profile, an appropriate statistical analysis investigating the non-monotonic nature of each dose-response relationship is most appropriate because it is based on individual results. So when an adequate amount of data is available, a statistical assessment is performed. An NMDR is considered plausible if the results are significant (p < 0.05). However, individual data are often too poorly reported in literature to conduct such statistical analysis although an NMDR might exist. For those cases, a specific scoring procedure is applied in a fourth step to assess the strength of the NMDR relationships. This scoring procedure is derived from the criteria proposed by Calabrese and Baldwin [12] for hormesis (a specific type of NMDR profile). Briefly, the procedure developed by Calabrese and Baldwin is a numeric scoring assignment value including the number of tested dose levels, the magnitude of the response associated with each dose compared to the basal level, the significance of the response at each dose, and the presence of other studies confirming these data (Table 1). These criteria were used because we considered these parameters important and sufficient to define the non-monotonic nature of a dose-response relationship and estimate the plausibility of a presumed NMDR relationship (Table 2). The criteria used here are the criteria suggested by Calabrese and Baldwin adjusted for a use in this application. Each score was assessed individually without considering whether NMDR relationships were reported or not in the same study. A score of “moderate,” “moderate–high” or “high” was considered in this work as indicative of a sufficient strength. The fifth and last step of the assessment is to determine whether the previously suspected NMDR relationship is also supported by biological plausibility. So an additional assessment of the literature is made to check whether additional information would support the biological plausibility and then increase the confidence in the reported NMDR.

**Table 1:** **Summary of criteria of analysis with assigned point values used in the evaluation of statistical plausibility of an NMDR relationship**

Number of doses below ZEP (excluding the control)		Score A
1		1
2		2
3		3
4		4
≥5		5
**Experimental determination of ZEP**		**Score B**
Yes		1
No		0
**Number of doses statistically different from control**		**Score C**
1		2
2		4
3		8
≥4		16
**Reproducibility of the dose-response relationship**		**Score D**
Yes		3
No		0
**Magnitude of response (percentage control value)**		**Score E** ^**a**^
**Inverted-U curve**	**U curve**	
≥100%, ≤125%	≤97%, ≥92%	0,5
>125%, ≤150%	<92%, ≥84%	1
>150%, ≤200%	<84%, ≥68%	2
>200%, ≤400%	<68%, ≥5%	3
>400%	<5%	4

^a^The point value is multiplied by the number of experimental doses falling within the corresponding percentage range.

Criteria reported (Table 1) were extracted from Calabrese and Baldwin [12]. The ZEP (zero equivalent point) corresponds to the point where the response crosses the control value in a hormetic effect.

**Table 2:** **Summary of total score for plausibility of an NMDR relationship**

Total score ^a^	Plausibility of an NMDR relationship
1–2	No–low
>2–8	Low
>8–12	Low–moderate
>12–16	Moderate
>16–20	Moderate–high
>20	High

^a^These scores were extracted from Calabrese and Blain (2011) [14].

The biological plausibility of NMDR relationships reported as significant, or scored to have sufficient strength, is evaluated by considering whether mechanistic explanations are proposed or demonstrated for the observed dose-response relationship: (1) the NMDR relationship is explained or not by high-dose toxicity (e.g., cytotoxicity) or physico-chemical effects, (2) a known or proposed mechanism of action that could explain the observed effect is described, or (3) the existence of *in vivo*, *in vitro,* or *in silico* experimental data that support a new mechanism of action. If the proposed hypotheses are supported by experimental evidence, then the biological plausibility is considered as reinforced. In this case, the study is considered as conclusive and qualified useful for risk assessment. If it is concluded that there is a lack of biological plausibility in the study or that a mechanistic explanation or a hypothesis is not developed by the authors, the NMDR is scored as a suspected NMDR, needing either further testing or mechanistic studies.

Lastly, the developed stepwise decision tree is applied to a case study focused on BPA *in vivo* studies showing NMDR. This case study was selected because the literature search results identified several *in vivo* studies depicting or claiming NMDRs with this compound and because of the particular interest of the ANSES Working Group on Endocrine Disrupters and on BPA in particular, which was under evaluation by this committee.

## Results

### Literature review of studies describing NMDR relationships

The first step of this analysis was to perform a targeted literature search, which identified 219 publications in the PubMed database up to January 2012. Of these publications, 51 experimental studies described one or more NMDR profiles concerning EDCs and/or natural hormones. Out of these 51 studies, 2 were epidemiological studies [15, 16], 20 were in vitro studies [6, 17–35], and 29 were *in vivo* studies [36–64]. From these 51 studies, 170 dose-effect relationships were claimed by the authors as NMDR, most of them involving an endocrine disruption (Table 3). The most often-cited substances were BPA and 17β-estradiol (E_2_).

**Table 3:** **EDCs and the number of associated NMDR relationships**

Substance		Number of NMDR relationships
**Hormone**		**42**
	17β-estradiol	35
	17α-estradiol	2
	Ethinyl estradiol	2
	Dihydrotestosterone	1
	Pregnenolone	1
	Dehydroepiandrosterone	1
**Bisphenol A**		**60**
**Alkylphenols**		**16**
**Diethylhexylphthalate**		**10**
**Phytoestrogens**		**10**
	Coumestrol	2
	Daidzein	2
	Genistein	3
	Lavendustin	1
	Resveratrol	2
**Pesticide**		**12**
	Organochlorine	9
	Methoxychlor	3
**PCBs**		**9**
**Diethylstilbestrol**		**12**

The NMDR profiles were reported for both animal sexes, for various physiological and behavioral effects, and for several organs or systems (Table 4). Affected organs and systems included the central nervous system, hypothalamic-pituitary axis, mammary glands, adrenal glands, the cardiovascular system, and the male and female reproductive systems. In 4 studies [26–29], the effects having the highest occurrence were related to modulation of prolactin release (12 NMDR relationships) and changes in protein kinase activity (11 NMDR relationships) in pituitary cells. A modification of the mammary structure in mice (e.g., numbers and volume of terminal end buds and density of the mammary epithelium) was reported in 6 publications in which 15 NMDR profiles were identified [39, 47, 57, 58, 60, 61]. It is important to note that some NMDR relationships observed with BPA and E_2_ were related to modes of action mediated by estrogen receptors and rapid signaling mechanisms. These effects include the release of prolactin and the phosphorylation of protein kinases in pituitary cells, cardiomyocyte contractility, structural modifications of mammary terminal end buds, and modification of epididymal weight. Additionally, the epidemiological studies identified 8 NMDR profiles for metabolic effects associated with endpoints such as increased body mass index, insulin resistance, altered triglycerides and high-density lipoprotein (HDL) levels, and increased risk of soft-tissue sarcoma.

**Table 4:** **The effects associated with NMDR relationships**

Organs and biological functions involved		Observed effect (with number of associated NMDR relationships) ^a^		Substances involved
		***In vitro*** studies	***In vivo*** studies	
Hypothalamus		Dopaminergic transmission (2)	Aromatase activity in preoptic area (1)	E2, octylphenol, diethylhexylphthalate (DEHP)
Pituitary gland		Cell proliferation (1) GABAergic transmission (2) Calcium channel activity (2) Luteinizing hormone (LH) release (1) Prolactin release (12) Protein kinase modification (11)	Follicle stimulating hormone (FSH) plasma level (1) LH plasma level (1)	E2, phytoestrogens, BPA, alkylphenols, DES, organochlorines
Male reproductive system	Testes	Cell proliferation (1) Spermatid DNA breaks (1)	Weight (2) Testosterone hydroxylase activity (4)	E2, BPA, ethinyl estradiol
	Epididymis,		Weight (2)	E2, BPA
	seminal vesicle,		Weight (1)	BPA
	preputial glands,		Weight (1)	BPA
	prostate	Cell proliferation (1)	Weight (5)	E2, BPA, DES, dihydrotestosterone (DHT), alkylphenols
Female reproductive system	Ovary	Progesterone secretion (1) Testosterone secretion (1) Estradiol secretion (1)	Transcriptional activity (1)	PCBs, BPA
	Uterus		Percentage of epithelial cells ERα + (1) Progesterone receptor expression (1)	BPA
Breast			Modification of mammary epithelium and terminal end buds (15)	E2, BPA, DES
Brain		L-prostaglandin synthase activity (2) Calcium channel activity (2)		E2, pregnenolone, dehydroepiandrosterone (DHEA)
Cardiovascular system	Heart	Myocyte contractility (3)		E2, BPA
	aortic smooth muscle	Cell proliferation (1) Intracellular pH (1) Modification of protein kinase (2)		E2, BPA
Adrenal glands			Corticosterone plasma level (1)	PCBs
Liver			Nuclear receptors expression (5) Transcriptional factors expression (4)	BPA
Perinatal development			Age of puberty (1) Number of newborns (2) Weight of newborns (4) Anogenital distance (3) Sex-ratio (2) Newborn viability (3) Femur length (1) Growth (1)	BPA, DEHP, DES, ethinyl estradiol, alkylphenols, methoxychlor, organochlorines
Behavior			Immobility period (1) Spatial memory (2) Temporal memory (2) Nocturnal activity (1) Territorial urine-marking (1) Sexual behavior (5)	E2, 17α-estradiol, BPA, DES
Metabolism	Lipids	Adiponectin release (2) Adiponectin expression (1)	Triglycerides levels (1) Lipogenesis gene expression (10) Cholesterol metabolism gene expression (4)	BPA
	Glucose		Glucose metabolism gene expression (2) Insulin levels (1)	BPA
Immunity		Mast cells degranulation (4)	Rate of degranulated eosinophils and last cells (1) Macrophagic activity (1) Severity of allergic skin lesions (1)	E2, organochlorines, alkylphenols, DEHP
Cancer			Tumor multiplicity (1) Tumor volume (1) Latency period for first tumor (1) Percentage of lung metastases (1)	BPA

^a^Numbers in parentheses represent the number of NMDR relationships associated with each corresponding effect.

#### Quality analysis of NMDR relationships

To evaluate the plausibility of the NMDR profiles observed, a series of criteria was applied based on those developed by Calabrese and Baldwin [12]. The first criterion was the minimal number of dose tested levels. To be considered acceptable, in addition to control group to establish endpoint baseline, a minimum of 3 dose levels were required.

NMDR relationships were reported in 2 epidemiological studies [15, 16]. Both studies were analyzed to assess the plausibility of the dose response relationship reported by the authors. Regarding the study from Tuomisto et al. [15], the scoring criteria procedure could not be applied due to data inconsistencies. It was concluded that the available data were not appropriate for scoring. This conclusion was reached because the exposure range was treated inconsistently and in a manner that resulted in diverging conclusions regarding the shapes of reported dose-response curves. For example, in the case of dioxins, when the analysis was performed with the World Health Organization’s toxicity equivalent (WHO-TEQ) expressed in septiles, the soft tissue sarcoma risk was higher in the lowest septile than in the other septiles, and the differences from control was significant in the second and the sixth septiles. However, when the analysis was performed with WHO-TEQ expressed in quintiles, the odds ratios were not significantly different. Moreover, when confounders such as sex, age, or education were included in the analysis, the odds ratios were decreased in a linear manner. For those reasons, it is unclear whether the observed, decreasing trend of soft tissue sarcoma risk with increasing exposure to dioxin is non-monotonic. Regarding the study from Lee et al. [16], many significant associations of persistent organic pollutants (POPs) with dysmetabolic conditions appeared at a low dose range, suggesting an inverted U-shaped dose-response relationship. As a result, a quadratic relationship was considered in specific statistical tests that supported the significance of several endpoints for some POP exposures. Examples of these exposures for high density lipoproteins (HDL) cholesterol level include p,p’-dichlorodiphenyldichloroethylene (DDE), polychlorobiphenyls (PCB-170, and PCB-206). Examples of these exposures for triglyceride levels include p,p’-DDE, oxychlordane, and trans-nanochlor. Based on the statistically significant results of the reported NMDR relationship, it was then unnecessary to use the criteria analysis derived from Calabrese and Baldwin [12]. Decision not to further consider this study was made mainly because of the categorical classification of exposure which results in the ability to only associate a dose range, rather than a specific exposure, to an observed outcome. Therefore a decision was made not to include a detailed and specific assessment of these two epidemiological studies.

Of the 162 NMDR profiles reported in the selected *in vitro* and *in vivo* studies, 14 were rejected for at least one reason (number of tested doses less than 4, absence of control, no individual numerical values, or absence of statistical tests assessing the significance of the differences between measured values). In some studies (e.g. [39, 46, 49, 60]), the authors documented specific statistical analyzes that were considered to be valid or to satisfy criteria for non-monotonicity. The dose-response relationships were therefore considered having a high plausibility for non-monotonicity. In the other studies reviewed, no specific statistical analysis was used to verify the non-monotonic nature of the dose-response relationships. The remaining 148 NMDR relationships were then analyzed using the adapted scoring criteria (Table 5). Out of those 148 reported NMDR relationships, 82 were concluded to have moderate, moderate–high, or high plausibility of being non-monotonic. There were no NMDR profiles classified in the “no–low” plausibility category, mainly because dose-effect relationships identified from studies with only three tested doses, including the control, were *a priori* rejected and unscored.

**Table 5:** **The statistical plausibility of NMDR relationships**

Plausibility of NMDR relationships	n ( ***in vitro*** )	n ( ***in vivo*** )	n (total)
No–low	0	0	0
Low	9	29	38
Low–moderate	9	20	29
Moderate	9	17	26
Moderate–high	6	10	16
High	20	20	40

Note: n = the number of NMDR relationships.

#### Biological plausibility analysis

In 20 out of the 51 experimental studies, no mechanistic hypothesis for the observed NMDR relationships was proposed by the authors. In the 31 remaining studies, the authors suggested multiple physiological mechanisms to explain the observed effects. Proposed mechanisms or mode of action included actions at several molecular targets (e.g., receptors, ion channels and signaling proteins) with differing affinities for the substance, the induction of antagonistic effects, negative feedback regulation to reduce responses, or receptor desensitization. Additional factors proposed as possibly responsible for NMDR relationships included dose-dependent metabolism modulation, high dose toxicity, and/or a dose-dependent protein ionization to generate chaotropic modulation of activity (Table 6).

**Table 6:** **Mechanistic hypotheses reported in corresponding studies**

Mechanistic hypotheses	References
Existence of several molecular targets with different affinities and opposite effects	[6, 16, 18, 19, 24, 26–28, 34, 38, 42, 43, 45–47, 50, 54, 55, 57, 58, 60, 61, 63]
Negative feedback phenomenon	[6, 25, 31, 36, 47, 50, 57, 58, 60, 61, 63]
High-dose receptor desensitization	[25, 44, 52]
Dose-dependent metabolism modulation	[16, 38]
High-dose toxicity	[41, 47, 49]
Dose-dependent protein ionization	[35]

**Figure 2:**
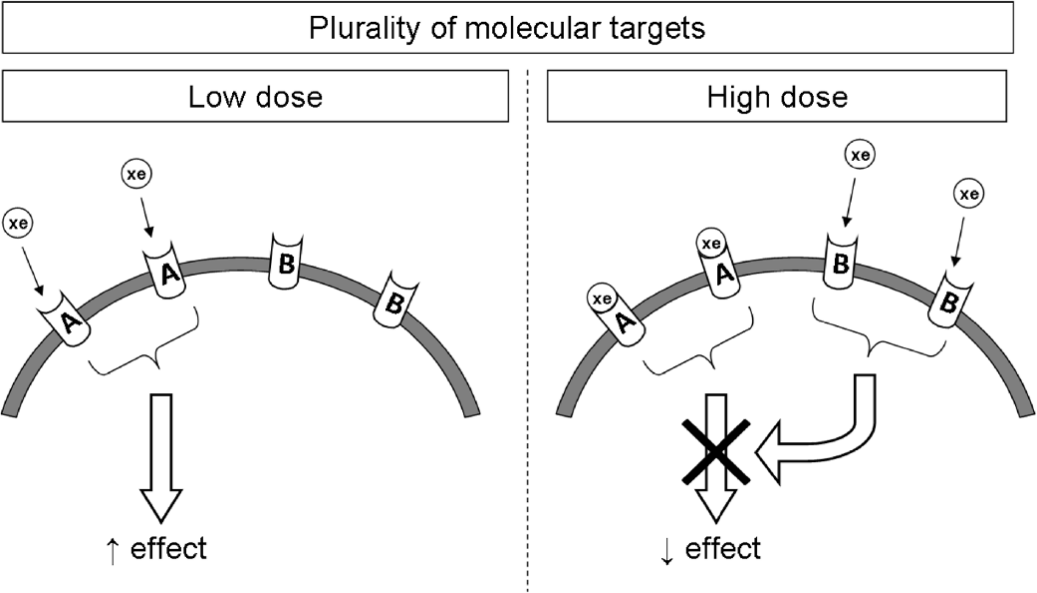
**Mechanism of the NMDR relationship phenomenon induced by the “plurality of molecular targets”.** At low concentrations, EDC binds to the A receptors and induces the observed effect. At high concentrations, the A receptors are still activated and EDC binds to the B receptors, which induces the opposite effect, resulting in an NMDR. Notes: A = Receptor A; B = Receptor B; xe = xenobiotic (e.g., EDC); affinity for A > B.

The most frequent hypothesis proposed to explain NMDR profiles was related to induction of opposing effects (e.g., agonist versus antagonist) across the range of the tested doses. These effects could be initiated by several molecular targets (predominantly receptors) that could be differentially activated by the same substance at different concentration levels and would be dependent on the affinity of the targets for the substance (Figure 2). This differential activation could be exemplified by several studies conducted on substances such as BPA, E_2_, phytoestrogens, diethylstilbestrol [DES] with known differences in affinity for ERα and ERβ, which can induce opposing effects in various cells, tissues, or organs at specific concentration levels [6, 18, 27, 43, 60, 61]. Moreover, two studies described an altered balance between proliferative and proapoptotic effects potentially arising from differential receptor activation at different doses [16, 45].

Another commonly proposed hypothesis to explain an observed NMDR was negative feedback regulation mechanisms related to physiological endocrine control of hormone actions [65, 66]. However, in most of the studies identified, the authors considered that other unknown factors, in addition to negative feedback regulation, were necessary to result in the observed dose-response relationships.

**Figure 3:**
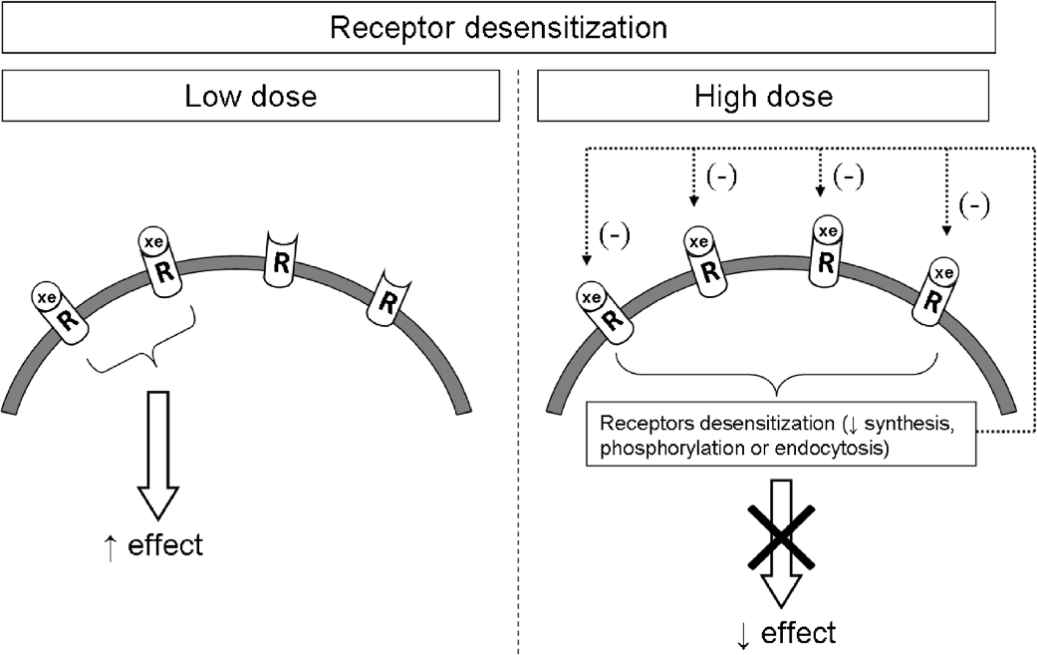
**Mechanism of the NMDR phenomenon induced by “receptor desensitization”.** At low concentrations, EDC binds to some receptors and induces the observed effect. At high concentrations, numerous receptors are bound, resulting in a down-regulation phenomenon characterized by receptor desensitization. Consequently, the intensity of the effect is decreased, resulting in an NMDR. Note: (-) = negative effect; R = receptor; xe = xenobiotic (e.g., EDC).

Receptor desensitization could also explain NMDR relationships (Figure 3). This phenomenon generally results from a mechanism involving protein phosphorylation, endocytosis, or repression of target receptor expression, leading to decreased receptor activity and insensitivity of cells/tissues to ligands at higher dose [67, 68]. Three studies suggested that desensitization, presumably through decreased expression of target receptors (estrogen receptors) after administering a high dose of substance (i.e., DES or E_2_), was responsible for the observed NMDR [25, 44, 52]. Time-dependent receptor desensitization is well established for gonadotropin-releasing hormone (GnRH), which is, at normal pulsatile low concentrations, required for fertility. Higher concentrations of synthetic GnRH agonists initially stimulate testosterone levels, but over time the level of plasma testosterone drastically decreases, resulting in infertility [69]. However, in the previously mentioned studies, specific experiments to address the validity of a mechanism involving time-dependent receptor desensitization or down regulation were not performed.

**Figure 4:**
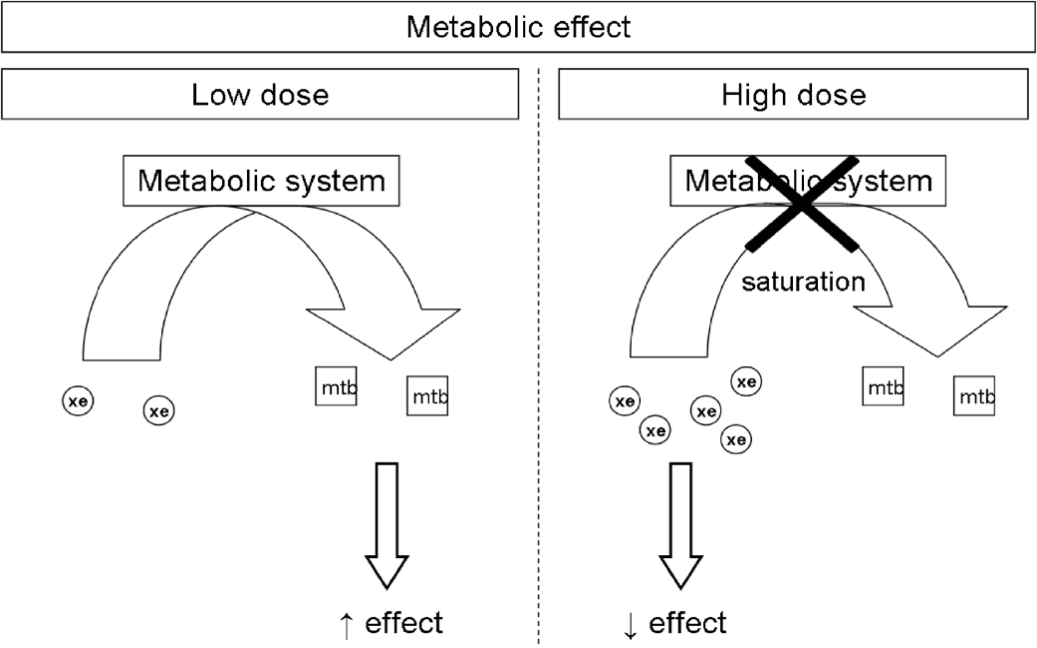
**Mechanism of the NMDR relationship phenomenon induced by one of the “metabolic effect” hypotheses.** At low concentrations, EDC is catabolized into active metabolites that induce the observed effect. At high concentrations, the metabolic system is saturated, and the parent substance induces an opposite effect, resulting in an NMDR relationship. Note: Mtb = metabolite; xe = xenobiotic (e.g., EDC).

**Figure 5:**
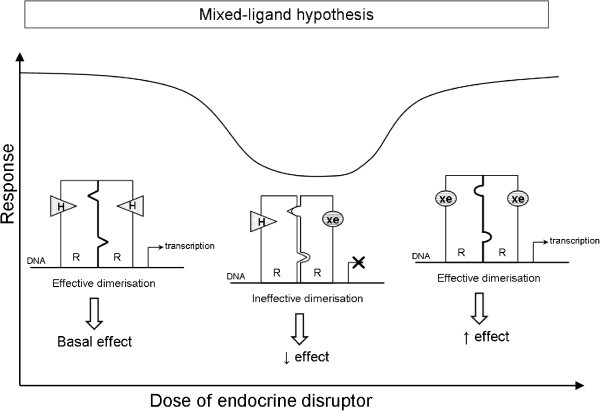
**Mechanism of the NMDR relationship phenomenon induced by the “mixed-ligand” hypothesis.** At low concentrations, the EDC binds to the hormone receptor and forms mixed-ligand dimers that block endogenous hormone activity. At high concentrations, dimers of EDCs are more likely to form and induce a response. Note: H = endogenous hormone; R = hormone receptor; xe = xenobiotic (e.g., EDC).

NMDR relationships may also be caused by metabolic modulation (Figure 4), as suggested in 2 studies [16, 38]. Modulation of gene expression due to formation of mixed-ligand dimers of steroid hormone receptors was also proposed to explain an observed NMDR (Figure 5) [70].

### Stepwise decision tree: case study on BPA

To consider all criteria in a systematic way, the stepwise decision tree previously described in the Material and Methods section (Figure 1) was applied to each selected NMDR profile reported for 10 BPA *in vivo* studies reporting NMDR curves. From these studies, 49 NMDR relationships were identified. There were many effects reported in these studies following exposure to BPA. These effects included an alteration of the response of mammary gland to progesterone [39] and increased mammary gland sensitivity to estrogens in mice [71] and increase in mammary tumorigenesis and metastasis in transgenic mice [45]. These effects also included alterations of uterine morphology and expression of estrogen and progesterone receptors in mice [40] and decreased fertility and fecundity in mice [72]. Additional effects included a deficit in the sexual behavior of male rats [46] and alterations in liver expression of several genes involved in lipogenesis [50]. The proposed decision tree could have been used to analyze NMDR relationships observed in *in vitro* studies. However, since *in vitro* studies were usually not selected as key studies for quantitative risk assessments, it was therefore decided to focus this analysis on *in vivo* studies.

#### Step 1: study quality assessment

The Klimisch score was used as an ancillary approach to evaluate the quality of the reported experimental data [13]. Klimisch categories 1, 2 or 3 are also assigned by the software-based tool “ToxRTool” (Toxicological data Reliability Assessment Tool). This tool was developed within the context of an ECVAM funded project to provide comprehensive criteria and guidance for evaluations of the inherent quality of *in vivo* and *in vitro* toxicological data (based on study reports or peer-reviewed publications). As a result, only the dose-responses of studies for which the assigned criteria was Category 1 (reliable without restrictions) or Category 2 (reliable with restrictions) were considered further. None of the retrieved studies followed the Organization for Economic Co-operation and Development’s (OECD’s) guidelines and/or complied with Good Laboratory Practices. However, because the protocols were well described and results were reported with adequate details, all the identified studies were considered to be Category 2 (reliable with restrictions) and were accepted for further analysis and scoring procedures.

A review of the protocol description indicated that specific parameters related to the external contamination of BPA to the laboratory animals were well controlled in some studies. For example, in the studies of Ayyanan et al. [39], Berger et al. [40], or Jones et al. [46], the mice were bred in a BPA–free environment using polysulfone or polypropylene cages and glass bottles. In the studies of Cabaton et al. [72], the presence of substances that may exhibit estrogenic activities in cages, food, water, and bedding was tested and found to be negligible. In the study of Jenkins et al. [45], the mice were fed phytoestrogen-free food, housed in polypropylene cages, and provided with water in glass bottles. In the study of Marmugi et al. [50], the authors reported using a “standard diet,” and no information was provided about the housing conditions, which was considered to be shortcomings [73].

#### Step 2: number of tested doses (including control)

In the studies of Adewale et al. [36] and Vom Saal et al. [64], the authors tested only 2 doses and a negative control group. Therefore, no further assessment of these 5 reported NMDR relationships from those studies was possible. For all other *in vivo* studies, the number of tested doses varied between 4 and 8, including the negative control group. All NMDR profiles from those studies were scored.

#### Step 3: availability of adequate data for specific statistical assessment

In some studies, *a posteriori* tests performed by the authors such as the Bonferroni multiple comparison test [45, 71] or analysis of variance (ANOVA) were used to make comparisons between control and test groups or to determine whether obvious NMDR relationships exhibited a significant quadratic polynomial component [39, 40, 46]. For some studies, sufficient data were not available for specific statistical evaluation of the plausibility of an NMDR profile. In those situations, the analysis criteria adapted from Calabrese and Baldwin was used to provide enough confidence to grade the plausibility of NMDR without a statistical test.

#### Step 4: criteria analysis procedure

On the basis of criteria derived from those of Calabrese and Baldwin, 44 *in vivo* NMDR profiles were analyzed. In one study [45], the available data did not allow us to apply the analysis criteria for one endpoint (time to first tumor latency) and thus the scoring procedure was not performed. Finally, the criteria for 43 *in vivo* NMDR were applied. Out of these 43 relationships, 23 had a no–low or low-moderate plausibility of being non-monotonic, and 20 had moderate or high and very high likelihood. On the basis of the scoring criteria, the studies in which obvious NMDR could be considered for further analysis of plausibility were those of Jenkins et al. [45], Jones et al. [46], and Marmugi et al. [50].

#### Step 5: biological plausibility

The authors of the publications on the 20 dose-response relationships scored with moderate to high and very high plausibility of being non-monotonic proposed two mechanistic explanations: a plurality of molecular targets and/or negative feedback regulation. Jenkins et al. [45] investigated the potential mechanisms behind the NMDR profile. The data suggested that the NMDR profile observed for tumorigenesis was due, at least partially, to the differential ability of BPA to induce apoptosis at each dose. Thus, at high doses of BPA, apoptosis would counter-balance cell proliferation that contributed to tumorigenesis at low doses. Jenkins et al. provided experimental data to support that hypothesis. Jones et al. [46] considered the ability of BPA to bind different types of receptors, such as ERα, ERβ, androgen receptors or thyroid hormone receptors with different affinities that may explain the NMDR profile observed in male rat sexual behavior. Jones et al. also mentioned the epigenetic activity of BPA as a possible mode of action. However, even if these mechanisms were considered relevant modes of BPA action, no specific experimental data was generated to support these hypotheses for these effects, and then must be considered speculative. In the study of Marmugi et al. [50], molecular mechanisms underlying the observed responses were explored and non dose-related expression of genes involved in lipid metabolism could support their observations. Finally, only the studies of Jenkins et al. and Marmugi et al. could be considered for risk assessment. Whereas the study of Jones et al. could be considered in a risk assessment process if the hypothesis for mode of action is confirmed by further testing.

## Discussion

The purposes of this work were to review the literature to identify NMDR relationships observed for some EDCs, to develop a methodology to assess whether those dose-response relationships were sufficiently reliable for use in risk assessments. In this qualitative approach, a judgment on the quality of the reviewed studies was not introduced, except for the case study on BPA. Rather, the aim was to derive tools that allow consideration of NMDR relationships in risk assessments. In recent years, NMDR profiles have been reported in the literature with an increasing frequency from a variety of *in vitro* and *in vivo* toxicological models involving substances that affect hormonal systems [10, 74, 75]. In an extensive review by Vandenberg et al. [10], they reported hundreds of examples of possible NMDR relationships for more than 20 natural hormones and more than 70 putative EDCs. Those examples were from studies performed in cultured cells, on whole animals or on human. It was not a surprise that such relationships were observed because of the complexity resulting from the many modes of action through which EDCs may influence the actions of hormones. Along with the potential for complex pharmaco/toxicodynamic influences (e.g., expression of varying levels of multiple receptors and possible interactions with native ligands), the critical feedback mechanisms involved with regulation of hormonal systems creates a level of increased complexity. Dose-response relationship for EDCs would reflect this complexity and would likely result in a non-monotonic dose-response. Studies using several hormone-sensitive cell lines have shown that NMDR relationships can result from a variety of mechanisms [9, 76]. However, that type of relationship is not exclusive to EDCs and is also observed for chemicals substances that do not act on the endocrine system, and can be elicited by non-chemicals stressors, such as ionizing radiations [7, 77].

This work intentionally considers the *in vivo* and the *in vitro* studies in which the authors of identified publications claimed that the dose-response relationships observed during their experiments had an NMDR profile. Here, the aim was not to compare monotonic dose-response relationships with NMDR profiles, but to evaluate the plausibility of non-monotonicity when assessing the dose-response relationship from all available data. Indeed, NMDR profiles are still a controversial issue, especially regarding their usefulness for risk assessments [3, 78].

In this current study, 51 experimental studies describing 170 NMDR profiles concerning EDCs and/or natural hormones were selected. Out of these 51 investigations, the two epidemiological studies [15, 16] were excluded since the scoring criteria procedure was not applied on them.

For the risk assessment point of view, the reproducibility of the effects displaying NMDR profiles should be taken into account because it supports the validity of an observed relationship, and improves the confidence in the biological observations reported. Considering reproducibility of dose-response relationships, most of the *in vivo* studies reporting NMDR profiles did not lead to further experiments by other scientific teams which prevent to assess their reproducibility. When studies appear to have been replicated, there were often variations in the experimental designs. This can be illustrated by the *in vitro* studies of Kochukov et al. [28] and Wozniak et al. [34] that both show U-shaped relationships for the effects of BPA on prolactin release from rat pituitary cells. However, due to differences in experimental systems, differences in the shape of the resulting concentration-response curves are observed that result in major differences in the apparent potency of BPA.

To some degree, the observation of a NMDR for a compound at different endpoints can increase confidence in the validity of NMDR relationship. In the Marmugi et al. study [50], 18 NMDR profiles related to different metabolic endpoints were reported: regulation of plasma insulin (1 NMDR relationship) and changes in gene expression related to lipid biosynthesis (17 NMDR relationships). In a weight of evidence approach, these observations are considered with lower score compared to NMDR relationships reported from 2 independent studies. It was not the aim of the current study to collect and compare all of the available measurements concerning one compound for the same endpoints. The literature search, as part of the current study, focused only on studies claiming NMDR relationships, which did not afford the opportunity to retrieve studies that do not report NMDR relationships. Therefore, it is currently unfeasible to establish with confidence the degree to which specific NMDRs are reproducible.

The scoring assessments used for this current study have been adapted from those previously used by Calabrese and Baldwin and Calabrese ans Blain [12, 14] for the quantitative identification of hormesis. We consider these criteria to be a useful starting point to develop a more general approach for analysis of NMDR relationships. As a result of the current study, we have identified some possible limitations of the approach in its current form. For example, the zero equivalent point (ZEP) criteria (Scores A and B) is an arbitrary definition applicable to hormetic responses, which requires reversal of the response and a complete return to the control value. However, this value is not always observed or even expected for EDCs. Future efforts should be made to elaborate more specific criteria that are independent, but inclusive of hormesis, that would result in a generalizable approach. Additionally, it will be necessary to develop a scoring criterion that considers dose range and the number of dose levels.

The specific nature (curve shape) of an NMDR relationship observed from a toxicological assessment has important ramifications for the risk assessment. In different cases, NMDR may be observed in concentration ranges for which the effect is still adverse. Thus, U-shaped dose-effect relationships could be observed with maximal effects at the extreme doses of the U-shape. In this case, it may be expected that at a dose far lower than the lowest tested dose levels, the effect will decrease again, because infinite toxicity cannot be observed at dose levels close to the zero dose. Conversely, bell-shaped dose-effect relationships may be observed with minimal effects induced by the two extreme doses that are not equal to the control response. In this case, at very high doses, unspecific effects will, for example, appear because the organism would be completely overwhelmed by the substance. Thus, it appears credible that U-shaped and bell-shaped dose-response relationships might correspond to two parts of a same dose-response relationship. At high doses, the substances could impair biological processes by non-specific mechanisms (e.g., choatropic effect, membrane disruption, unspecific binding to proteins). However, because a complete profile is rarely available, we are proposing two ways to use NMDR in risk assessments. For bell-shaped profiles, a NOAEL or a benchmark dose could be extrapolated by modeling the ascending part of the dose-effect relationship. For a U-shaped profile, it could be recommended to experimentally verify, or to model, whether the exposure levels are in the same range of doses for which an NMDR relationship is observed. The main improvement in the testing strategy would be to conduct a better investigation of the dose-response relationship by testing more doses, especially in the lower dose range, as it was proposed during the workshop on “The low dose effects and non-monotonic dose responses for endocrine active chemicals: Science to practice”, held in Berlin, Germany, on September 12–14^th^, 2012 [79]. These proposals could be included in the revision of OECD’s guidelines. If more dose groups are used, then the benchmark dose approach might be preferred because of the possible reduction in the number of animals per dose group. This approach was also proposed at the European Food Safety Authority’s 17^th^ Scientific Colloquium on Low Dose Response in Toxicology and Risk Assessment, held in Parma, Italy, on June 14 and 15, 2012 [2].

The relevant EDC modes of action that explain these NMDR relationships are still poorly investigated. Of the BPA studies fully considered, only 1 study explored the hypothesized mechanisms of action [44]. In a recent review from Vandenberg et al. [10], several mechanisms producing NMDR relationships were discussed, but for most of them only hypotheses were proposed which could be considered purely speculative, and their biological basis could not be fully assessed. Mechanisms involved in a non-monotonic profile are often described *in vitro*, but defining the *in vivo* mode of action could be much more complicated.

A significant biological plausibility of NMDR does not systematically imply the causality of adverse effects. For the purpose of risk assessments, the endpoints and the potential impact on human health must be considered when defining adversity. Determining whether an effect is adverse can be difficult for subtle or small-magnitude effects. In some situations, as previously mentioned, it would be reasonable to think that some of the effects characterized as presenting an NMDR might have a linkage to compensatory effects and not to endocrine disruption, leading to an adverse effect [7]. However, some of the endpoints identified in this study (Table 3) might be considered to be adverse. Moreover, adversity should also be considered regarding the exposure period. Indeed, there are windows of sensitivity in which specific subpopulations may be more vulnerable [80, 81]. In animals, EDCs have the potential to cause reproductive or developmental toxicity or several types of cancers in hormonally responsive organs such as the uterus or the mammary gland in females and the prostate in males [82, 83]. Thus, biological effects can be reversible or weakly severe in adults, but can be serious and damaging if exposures occur during specific critical life period (often prenatal or perinatal). For some substances, the NMDR profiles are observed exclusively when exposure occurs during a sensitive period of development (i.e., fetuses) when hormone-sensitive tissues such as uterine are most susceptible to the effects of EDCs. Consequently, during the fetal windows, low doses may impair physiological mechanisms responsible for NMDR relationships that do not exist during adulthood [9, 76, 81, 82].

## Conclusions

For this current work, we developed a methodology to consider individual NMDR relationships in a risk assessment context. We expanded a stepwise decision tree to assess the likelihood of NMDR relationships reported. This analysis can be completed with a weight of evidence approach to evaluate all the data available for the substance of interest. This approach enables assessing the consistency of the dose-response relationship. The steps in this approach involve assembling the relevant data (either positive or negative), evaluating these data for quality and relevance, and integrating the different points of evidence to support conclusions concerning specific properties of the substance. Thus, the relevance and the impact of NMDR relationships reported on EDCs can be better understood and incorporated into the risk assessment method to determine the potential impacts of such substances on human health. Another important point to determine is the extent of experimental exposure (the range of exposure level in which an NMDR relationship occurs). The literature review performed for this current study enabled an analysis of the NMDR relationship profiles reported by the authors.

## Abbreviations

ANOVA: Analysis of variance
BPA: Bisphenol A
DDE: Dichlorodiphenyldichloroethylene
DEHP: Diethylhexylphthalate
DES: Diethylstilbestrol
DHEA: Dehydroepiandrosterone
DHT: Dihydrotestosterone
E_2_: 17β-estradiol
EDC: Endocrine disrupting chemical
ER: Estrogenic receptor
HDL: High-density lipoprotein
FSH: Follicle stimulating hormone
GnRH: Gonadotropin-releasing hormone
LH: Luteinizing hormone
LOAEL: Lowest observed adverse effect level
NMDR: Non-monotonic dose-response
NOAEL: No observed adverse effect level
OECD: Organisation for economic co-operation and development
PCB: Polychlorinated biphenyl
POP: Persistent organic pollutant
WHO-TEQ: World health organization’s toxicity equivalent
ZEP: Zero equivalent point.
